# Attention-Deficit/Hyperactivity Disorder (ADHD)

**DOI:** 10.1192/j.eurpsy.2025.190

**Published:** 2025-08-26

**Authors:** S. C. Cortese

**Affiliations:** University of Southampton, Winchester, United Kingdom

## Abstract

**Abstract:**

Attention-Deficit/Hyperactivity Disorder (ADHD) In my talk, I will present the latest evidence, mainly based on previous meta-analyses, network meta-analyses, dose-response meta-analyses, and umbrella reviews, that can inform clinical decision-making in the field of pharmacological treatment for ADHD, including the choice of initial medication, titration, treatment of stimulant-refractory cases, management of cases with comorbid conditions, and management of adverse event.

**Disclosure of Interest:**

None Declared

